# A High‐Performance Liquid Chromatography Method for Simultaneous Analysis of Naringin, Ascorbic Acid, and Caffeine: Design, Development, and Validation

**DOI:** 10.1155/ianc/6287324

**Published:** 2026-02-13

**Authors:** Murad Abualhasan, Mohammad Mousa, Hitaf Izhiman, Malak Alkhatib, Ibaa Aghbar

**Affiliations:** ^1^ Faculty of Pharmacy, An-Najah National University, P.O. Box 7, Nablus, State of Palestine, najah.edu

## Abstract

The simultaneous quantification of ascorbic acid, caffeine, and naringin is of significant interest due to their potential therapeutic applications, particularly in the prevention and management of colorectal cancer. While numerous HPLC methods exist for the individual determination of each compound, our method stands out for its reliability in addressing all three compounds in a single analytical run. This study aimed to develop and validate a robust reverse‐phase HPLC method for the simultaneous analysis of these compounds in accordance with ICH Q2(R1) and USP guidelines. A robust reverse‐phase high‐performance liquid chromatography (RP‐HPLC) method was developed and validated for the simultaneous determination of ascorbic acid, caffeine, and naringin in the concentration range of 7.813–125 μg/mL. Chromatographic separation was achieved on a Symmetry Shield RP‐18 column (250 × 4.6 mm, 5 μm) using an isocratic mobile phase of 75% phosphate buffer (pH 3.5) and 25% acetonitrile at a flow rate of 1 mL/min, with detection at 220 nm. Method validation confirmed specificity, selectivity, and excellent linearity (*R* ^2^ ≥ 0.9981). Precision testing showed RSD% values well below 2% for both intraday and interday analyses. Accuracy studies demonstrated mean recoveries within the range of 98%–102% (ascorbic acid: 100.05%, caffeine: 100.33%, and naringin: 100.47%). Robustness testing indicated that small deliberate changes in the flow rate and pH had no significant effect on retention times (RSD% ≤ 0.71%). System suitability parameters, including tailing factor (≤ 1.38), theoretical plates (> 2000), and resolution (> 3.2), met the acceptance criteria. The developed method is reliable, reproducible, and suitable for routine quality control applications that require the synchronized quantification of these three bioactive compounds.

## 1. Introduction

In recent years, there has been a growing interest in naturally derived compounds with therapeutic potential, particularly those exhibiting antioxidant, anti‐inflammatory, and anticancer properties. Among these, ascorbic acid, caffeine, and naringin are commonly used in pharmaceutical formulations, dietary supplements, and functional foods. Their beneficial health effects, particularly in the prevention and management of colorectal cancer, have been extensively studied, either individually or in combination [1–4].

**FIGURE 1:**
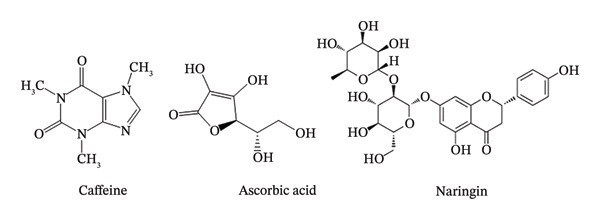
The chemical structure of caffeine, ascorbic acid, and naringin.

The chemical structure of ascorbic acid ((2R)‐2‐[(1S)‐1,2‐dihydroxyethyl]‐3,4‐dihydroxy‐2H‐furan‐5‐one) [5], caffeine (1,3,7‐trimethylpurine‐2,6‐dione) [6], and naringin ((2S)‐7‐[(2S,3R,4S,5S,6R)‐4,5‐dihydroxy‐6‐(hydroxymethyl)‐3‐[(2S,3R,4R,5R,6S)‐3,4,5‐trihydroxy‐6‐methyloxan‐2‐yl]oxyoxan‐2‐yl]oxy‐5‐hydroxy‐2‐(4‐hydroxyphenyl)‐2,3‐dihydrochromen‐4‐one) [7] is shown in Figure 1.

Ascorbic acid has been found to induce colorectal cancer cell death by increasing oxidative stress and through reactive oxygen species (ROS)–independent pathways [8–10]. Caffeine may reduce inflammation, thus contributing to the suppression of colorectal cancer progression [11, 12]. Naringin exhibits cytotoxicity against colon cancer cell lines with an IC_50_ of 63.14 μg/mL and promotes apoptosis via caspase‐3 activation, suggesting its promise as a chemopreventive agent [13, 14]. However, further investigation is necessary to elucidate its molecular mechanisms and determine optimal therapeutic doses [15].

Given the pharmacological importance of these compounds, accurate and reliable analytical methods are essential for their quantification in formulations, as demonstrated by pharmacokinetic and bioavailability studies for other bioactive molecule [16]. High‐performance liquid chromatography (HPLC) is considered one of the most effective techniques due to its high resolution, sensitivity, and reproducibility [17].

Sophisticated analytical techniques such as liquid chromatography coupled with mass spectrometry (LC‐MS/MS), GC‐MS, and other advanced spectroscopic methods enable the concurrent detection and quantification of combination compounds with high sensitivity and selectivity [18].

Although various HPLC methods have been developed for the individual analysis of these compounds, limited work has addressed their simultaneous determination using a single method [19]. For example, ascorbic acid has been analyzed using a mobile phase of 97% methanol and 3% water with a C18 column and detection at 254 nm [20]. Caffeine has been quantified using a mobile phase of 20% acetic acid and 80% water (pH 3), with a C18 column and detection at 254 nm [21]. Naringin was separated using a mobile phase of acetonitrile:water:formic acid (21:78.8:0.2) and detected at 280 nm with a C18 column [22].

There remains a lack of simple, cost‐effective, and fully validated chromatographic methods capable of simultaneously quantifying ascorbic acid, caffeine, and naringin in a single analytical run. Therefore, this study addresses this unmet need by introducing a novel, robust, and efficient HPLC method for the simultaneous quantification of ascorbic acid, caffeine, and naringin, offering a practical and accessible alternative for routine quality control analysis. The method was validated in accordance with ICH Q2(R1) guidelines and is suitable for routine analysis in quality control laboratories.

## 2. Methodology

### 2.1. Apparatus

The HPLC technique was utilized to analyze a mixture of ascorbic acid, caffeine, and naringin. A reverse‐phase chromatography analytical method was employed using a Waters 1525 binary HPLC pump, equipped with a six‐port manual injector and a Waters 2998 diode array detector. The Breeze 2 software was used for instrument control, data collection, and data processing. A nonpolar stationary phase column (Symmetry Shield RP‐18) was used for the analysis; this column measures 250 mm in length and 4.6 mm in inner diameter, packed with 5‐μm particles.

### 2.2. Standard Preparation

The stock solution of standards (1 mg/mL) was prepared by weighing 100 mg each of ascorbic acid, caffeine, and naringin. These were combined in a 100‐mL volumetric flask, to which 50 mL of a dilute solution consisting of 75% phosphate buffer (pH 3.5) and 25% acetonitrile was added. The mixture was thoroughly mixed and then diluted to volume with the same diluent. Serial dilutions of the stock solution were then performed to prepare standard solutions at concentrations of 125, 62.5, 31.25, 15.625, and 7.813 μg/mL.

### 2.3. Method Development

#### 2.3.1. Wavelength Selection

A mixed standard solution containing 31.25 μg/mL each of ascorbic acid, caffeine, and naringin was prepared in the mobile phase. The mixture was analyzed using an HPLC system equipped with a Waters 2998 diode array detector (DAD). A 20‐μL aliquot of the mixture was injected, with the flow rate maintained at 1.0 mL/min under the optimized chromatographic conditions.

Detection was carried out at four different wavelengths: 254, 243, 288, and 220 nm, by adjusting the detector setting while maintaining all other chromatographic parameters constant. For each wavelength, chromatograms were recorded, and the percentage peak area (% area) for each compound was calculated using the instrument’s data processing software. The results were compared to evaluate the relative detector response of each analyte and to determine the wavelength that provides the most balanced and sensitive simultaneous detection of ascorbic acid, caffeine, and naringin.

#### 2.3.2. Mobile Phase Selection

To select a mobile phase that provides the best separation in a shorter retention time (R.T.), multiple mobile phases were tested for their ability to achieve this separation, with two successfully separating ascorbic acid, caffeine, and naringin. A comparison was made between these mobile phases based on their run times.

### 2.4. Method Validation

Method validation is the process of demonstrating through laboratory studies that a specific analytical procedure consistently produces results that meet predefined criteria and are suitable for its intended purpose. The objective of this step is to confirm, according to the established validation guidelines and authoritative literature, that the method is reliable, accurate, and complete for its intended application (ICH Q2(R1), 2005; USP < 1225 >, 2020) [23, 24].

#### 2.4.1. Specificity and Selectivity

The expected components include ascorbic acid, caffeine, and naringin, and the method must demonstrate any potential interference from other substances present in the analysis. This method is specific for the analysis of all three components collectively or one component individually. Selectivity refers to the extent to which particular analytes in mixtures or matrices can be measured without interference from other components that exhibit similar behavior. The method demonstrates good separation in the HPLC chromatogram for the three components, as evidenced by distinct peak areas without any overlap, indicating a high level of selectivity. A mixture of the ingredients was injected into the system, and the resolution between the peaks was calculated [25, 26].

#### 2.4.2. Linearity

The linearity of the method was evaluated for ascorbic acid, caffeine, and naringin over a concentration range of 7.813–125 μg/mL. Standard solutions at five concentration levels within this range were prepared by serial dilution of stock solutions in the mobile phase.

Each solution was injected into the HPLC system under the optimized chromatographic conditions, and peak areas were recorded. The retention times for each analyte were monitored to confirm consistency across concentrations.

Calibration curves were constructed by plotting peak area (response) against the corresponding concentration for each analyte. Linear regression analysis was performed to determine the slope, intercept, and correlation coefficient (*R* ^2^) for each calibration curve. The acceptance criterion for linearity was a correlation coefficient of *R* ^2^ ≥ 0.995, according to international validation guidelines (ICH Q2(R1)) [27, 28].

#### 2.4.3. Precision

Instrument precision was evaluated by performing repeated injections (*n* = 7) of a standard mixture containing ascorbic acid, caffeine, and naringin at a concentration of 31.25 μg/mL. The retention times and peak areas were recorded for each analyte. The relative standard deviation (RSD%) was calculated to assess the repeatability of the instrument response—the generally accepted threshold of 2% specified in ICH Q2(R1) guidelines [23].

Intraday precision was assessed by analyzing the same concentration of each analyte (31.25 μg/mL) in triplicate at three different times within the same day (0, 3, and 6 h). Retention times and peak areas were recorded, and the RSD% was calculated.

Intermediate precision (interday) was determined by analyzing the analytes at the same concentration (31.25 μg/mL) over three consecutive days. Four replicate injections were performed daily, and retention times and peak areas were monitored. The overall %RSD values for each analyte over the three‐day period were <2%, confirming the method’s interday precision.

#### 2.4.4. Accuracy

The accuracy of the developed HPLC method was assessed through recovery studies using the standard addition technique. Known amounts of each analyte, ascorbic acid, caffeine, and naringin, were spiked into the sample matrix at three concentration levels corresponding to 0%, 12.5%, and 26.7% of the target test concentration (31.25 μg/mL). Thus, the total spiked sample will theoretically be 31.25, 35.16, and 39.6, respectively. Each spiking level was prepared and analyzed in triplicate to ensure reproducibility.

Samples were processed and injected into the HPLC system under optimized chromatographic conditions. Peak areas were recorded, and the percent recovery for each analyte was calculated by comparing the measured concentration to the theoretical spiked concentration using the formula:

(1) % recovery=measured concentrationtheoretical concentration×100.

The acceptance criteria for recovery and precision were based on ICH Q2(R1) guidelines, where the mean recovery should fall within the range of 98%–102% [29, 30].

#### 2.4.5. Robustness

Method robustness was demonstrated by the ability of the analytical procedure to remain unaffected by small, deliberate variations in method parameters. The robustness of the method was evaluated by assessing the impact of changes in mobile phase pH and flow rate [31, 32].

## 3. Results and Discussion

### 3.1. Result Wavelength

At a detection wavelength of 254 nm, ascorbic acid shows a very high % area, while caffeine has a low % area and naringin a very low % area, making this wavelength unsuitable for the synchronized detection of all three compounds.

At 243 nm, a similar trend is observed: ascorbic acid exhibits a very high % area, whereas caffeine and naringin show low and very low % areas, respectively. Therefore, 243 nm is also not optimal for simultaneous detection.

At 288 nm, ascorbic acid shows a very low % area, while caffeine and naringin both exhibit high % areas. This again limits the effectiveness of synchronized detection.

In contrast, at 220 nm, all three compounds demonstrate good % areas, making it the most suitable wavelength for synchronized detection of ascorbic acid, caffeine, and naringin. The detailed results are illustrated in Table 1.

**TABLE 1:** Percentage peak areas of the ascorbic acid, caffeine, and naringin mixture at different detection wavelengths​ (*λ*).

Ascorbic acid	Caffeine	Naringin
*Wavelength* (*λ*) *254 nm*		
75.4% area	21.34% area	3.23% area

*Wavelength* (*λ*) *243 nm*		
82.81% area	13.48% area	3.66% area

*Wavelength* (*λ*) *288 nm*		
10.7% area	40.24% area	48.98% area

*Wavelength* (*λ*) *220 nm*		
21.85% area	40.91% area	36.77% area

*Note:* Concentration of 31.25 μg/mL at 1 mL/min flow rate.

#### 3.1.1. HPLC Condition

**FIGURE 2:**
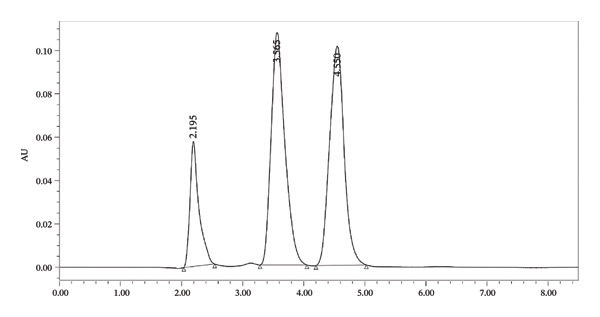
Chromatogram of ascorbic acid, caffeine, and naringin at 220 nm.

The mobile phase consists of isocratic elution using a mixture of 75% phosphate buffer (pH 3.5) and 25% acetonitrile. The stationary phase is a nonpolar C18 silica‐based bonded phase column (Symmetry Shield RP‐18), measuring 250 mm in length and 4.6 mm in inner diameter, packed with 5‐μm particles. The detector operates at a wavelength of 220 nm, with an injection volume of 20 μL for both the sample and standard. The mobile phase flow rate is set at 1 mL/min, and measurements are taken at ambient temperature (Figure 2).

### 3.2. Method Validation

#### 3.2.1. Result Linearity

The linearity concentration range for ascorbic acid, caffeine, and naringin was established from 7.813 to 125 μg/mL. The regression I e equation was as follows: *Y = *17,479*X + *34,764, correlation coefficient (*R* ^2^) = 0.9982; *Y = *49,610*X + *8018, *R* ^2^ = 0.9981; and *Y = *41,784*X + *48,941, *R* ^2^ = 0.9998 for ascorbic acid, caffeine, and naringin, respectively.

All test results meet the acceptance criteria outlined in the validation guidelines and reference texts, which require a correlation coefficient (*R* ^2^ ≥ 0.995). This indicates a strong linear relationship between the concentration and peak area (response) for ascorbic acid, caffeine, and naringin.

The retention times (R.T.) for ascorbic acid, caffeine, and naringin were consistent across five different concentration levels, further confirming the method’s reliability.

#### 3.2.2. Mobile Phase

During method development, several mobile phase compositions were systematically evaluated. Initially, mixtures of acetonitrile and water at different ratios were tested; however, these conditions resulted in inadequate peak separation among the analytes. Subsequently, methanol–water systems were investigated, but these also failed to provide satisfactory resolution and were associated with broad peak shapes and low theoretical plate numbers, indicating poor column efficiency. Based on these observations, these mobile phase systems were rejected, and further optimization was undertaken to achieve improved separation and chromatographic performance.

**FIGURE 3:**
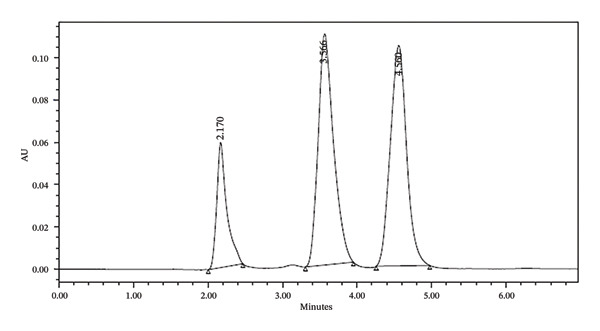
Chromatographic separation of the ingredient mixture using isocratic elution with 75% phosphate buffer (pH 3.5) and 25% acetonitrile.

The use of isocratic elution with 75% phosphate buffer (pH 3.5) and 25% acetonitrile resulted in significantly improved separation with shorter retention times for all analytes, enhancing method efficiency and reducing total run time (Figure 3). Moreover, the method gives good separation with short run time. The retention time and the peak separation are demonstrated in Table 2.

**TABLE 2:** Retention times of the ingredient mixture under different mobile phase compositions.

R.T. of ascorbic acid	R.T. of caffeine	R.T. of naringin
*Mobile phase 40%* (*methanol with 60% water*)		
3.118 min	6.968 min	33.485 min

*Mobile isocratic elution with 75% phosphate buffer* (*pH 3.5*) *and 25% acetonitrile*		
2.170 min	3.566 min	4.560 min

#### 3.2.3. Results of Precision

##### 3.2.3.1. Instrument Precision

The results of injecting the mixture on the same instrument to evaluate instrument precision showed consistent values for each compound. Both the retention times and peak areas exhibited minimal variation. For all three analytes, the RSD% values for retention time and peak area were well below the generally accepted limit of 2% specified in method validation guidelines (e.g., ICH Q2(R1)). This indicates that the instrument shows excellent precision when performing repeated injections under identical conditions. The detailed results are illustrated in Table 3.

**TABLE 3:** Instrumental precision for ascorbic acid, caffeine, and naringin.

*Instrument precision for ascorbic acid*		
Average	2.21	1128003.71
STD DEV	0.0169	6002.88
RSD (%)	0.7649	0.5321

*Instrument precision for caffeine*		
Average	3.601857143	3,116,193
STD DEV	0.026510555	14261.96
RSD	0.736024609	0.4576

*Instrument precision for naringin*		
Average	4.55	2,665,439
STD DEV	0.0076	7995.24
RSD (%)	0.1677	0.2998

##### 3.2.3.2. Intraday Precision

The RSD% values for retention time (0.1474%) and peak area (0.1335%) are well below the acceptable limit of 2% as per method validation guidelines (Table 4). Therefore, the intraday precision results for ascorbic acid meet the acceptance criteria. The RSD% values for retention time (0.1063%) and peak area (0.4104%) are within the acceptable limit of 2%. The intraday precision results for caffeine are therefore acceptable. The RSD% values for retention time (0.1376%) and peak area (0.1769%) are below the 2% limit specified in validation guidelines. Therefore, the intraday precision for naringin meets the acceptance criteria.

**TABLE 4:** Intraday precision for ascorbic acid, caffeine, and naringin.

Concentration 31.25 μg/mL	RT (min)	Area
*Intraday precision for ascorbic acid*		
Injection (0 h)	2.18	581,205
Injection (3 h)	2.18	579,969
Injection (6 h)	2.17	579,777
Average	2.18	580,317
STD DEV	0.0032	774.99
RSD	0.1474	0.1335

*Intraday for precision caffeine*		
Injection (0 h)	3.55	1,562,235
Injection (3 h)	3.56	1,559,061
Injection (6 h)	3.56	1,549,926
Average	3.56	1,557,074
STD DEV	0.0037	6390.53
RSD	0.10	0.4104

*Intraday precision for naringin*		
Injection (0 h)	4.53	1,376,438
Injection (3 h)	4.54	1,372,855
Injection (9 h)	4.55	1,371,804
Average	4.54	1,373,699
STD DEV	0.0062	2429.55

For all three compounds (ascorbic acid, caffeine, and naringin), the intraday precision results show RSD% values for both retention time and peak area well below the maximum acceptance limit of 2%. This confirms that the developed method demonstrates excellent intraday precision.

##### 3.2.3.3. Interday Precision

The aim of this step was to confirm that the method maintains precision during routine analysis when performed on different days. The study was carried out over three consecutive days at a concentration of 31.25 μg/mL for each analyte. Four replicate injections were performed daily, and the retention time was monitored through peak area measurements.

Across all three compounds, the RSD% values for each day and for the overall 3 day dataset were well below the 2% limit recommended by method validation guidelines (ICH Q2(R1)). This demonstrates that the developed method has excellent intermediate precision and is reliable for routine analytical applications performed on different days (Table 5). The interday precision results showed that the RSD% values for peak areas of ascorbic acid, caffeine, and naringin were all below 3%, meeting the acceptance criteria specified in method validation guidelines.

**TABLE 5:** Interday precision for ascorbic acid, caffeine, and naringin.

**Ascorbic acid (31.25 μg/mL)**	**Area 1** ^**st**^ **day**	**Area 2** ^**nd**^ **day**	**Area 3** ^**rd**^ **day**	**Average**

Injection 1	582,711	579,845	576,907	583417.25
Injection 2	583,401	579,806	577,845	579041.25
Injection 3	584,557	578,457	577,866	578018.75
Injection 4	583,000	578,057	579,457	577,148
Average	583417.25	579041.25	578018.80	580159.08
STD DEV	810.798115	920.31747	1057.997	2867.597267
RSD	0.1389	0.1589	0.1830	0.4942

**Caffeine (31.25 μg/mL)**	**Area 1** ^**st**^ **day**	**Area 2** ^**nd**^ **day**	**Area 3** ^**rd**^ **day**	**Average**

Injection 1	1,560,354	1,550,354	1,548,354	1563409.50
Injection 2	1,563,926	1,554,926	1,550,926	1552909.50
Injection 3	1,563,968	1,553,968	1,548,720	1,549,484
Injection 4	1,565,390	1,552,390	1,549,937	1,544,879
Average	1563409.50	1552909.50	1,549,484	1555267.667
STD DEV	2147.64	1998.92	1175.46	7256.073324
RSD	0.1373	0.1287	0.0758	0.466548201

**Naringin (31.25 μg/mL)**	**Area 1** ^**st**^ **day**	**Area 2** ^**nd**^ **day**	**Area 3** ^**rd**^ **day**	**Average**

Injection 1	1,378,986	1,378,986	1,365,908	1377759.75
Injection 2	1,379,904	1,372,904	1,366,465	1,375,652
Injection 3	1,376,543	1,375,438	1,365,422	1366768.75
Injection 4	1,375,606	1,375,280	1,369,280	1,374,505
Average	1377759.75	1,375,652	1,366,769	1373393.5
STD DEV	2018.30	2506.74	1727.54	5833.19
RSD	0.1464	0.1822	0.12639	0.4247

##### 3.2.3.4. ANOVA

A two‐way ANOVA without replication was employed to assess the effects of concentration and analyte type (ascorbic acid, caffeine, and naringin) on retention time (R.T.) and peak area (response). For retention time, the row factor (concentration range: 7.815–125 μg/mL) yielded a *p* value of 0.0841 (> 0.05), indicating no statistically significant effect of concentration. This confirms that retention times are independent of concentration, thereby supporting the precision and robustness of the method. In contrast, concentration had a statistically significant effect on the peak area, with a *p* value of 0.001, reflecting the expected proportional increase in response with increasing analyte concentration [33, 34].

#### 3.2.4. Accuracy

The method’s accuracy was evaluated by spiking known amounts of each analyte at three concentration levels (0%, 12.5%, and 26.7% of the test concentration 31.25) into the sample matrix. Thus, the total spiked sample will theoretically be 31.25, 35.16, and 39.6, respectively. Each level was analyzed in triplicate. The percent recovery was calculated using the measured concentration versus the theoretical spiked concentration.

Accuracy testing demonstrated mean recovery values of 100.05% for ascorbic acid, 100.33% for caffeine, and 100.47% for naringin, with all results falling within the acceptable recovery range of 98%–102% (Table 6). These findings confirm that the method exhibits excellent precision and accuracy for the determination of the three analytes under the tested conditions, making it suitable for reliable routine analysis.

**TABLE 6:** Accuracy of for ascorbic acid, caffeine, and naringin.

**Ascorbic acid**	**Conc (μg/mL)**	**Spike (μg/mL)**	**Total conc. (μg/mL)**	**Area**	**Recovery**	**(%) recovery**

Average	31.25	0	31.25	580879.5	31.24494	99.98382
		3.906	35.16	655516.25	35.51514	101.0101
		8.35	39.6	721,167	39.27122	99.16975

**Caffeine**	**Conc (μg/mL)**	**Spike (μg/mL)**	**Total conc. (μg/mL)**	**Area**	**Recovery**	**(%) recovery**

Average	31.25	0	31.25	1514637.25	31.41213	100.5188
		3.906	35.16	1708348.75	35.3168	100.446
		8.35	39.6	1920653.75	39.59626	99.99057

**Naringin**	**Conc (μg/mL)**	**Spike (μg/mL)**	**Total conc. (μg/mL)**	**Area**	**Recovery**	**(%) recovery**

Average	31.25	0	31.25	1375904.5	31.49377	100.7801
		3.906	35.16	1538086.75	35.39192	100.6596
		8.35	39.6	1,712,618	39.58688	99.96686

#### 3.2.5. Results of Robustness

The robustness study was performed for ascorbic acid, caffeine, and naringin by introducing small deliberate variations under chromatographic conditions: flow rate (±0.05 mL/min) and mobile phase pH (±0.05). Across all tested conditions, the RSD% of retention time remained well below the acceptance criterion of ≤ 3% specified in validation guidelines. Specifically, RSD% values for ascorbic acid ranged from 0.21% to 0.71%, for caffeine from 0.44% to 0.69%, and for naringin from 0.10% to 0.16% (Table 7). These results confirm that minor variations in the flow rate and pH do not significantly affect the retention time reproducibility, demonstrating that the developed method is robust for the simultaneous determination of all three analytes.

**TABLE 7:** Robustness results for ascorbic acid, caffeine, and naringin.

**Ascorbic acid**			
**Flow rate: 0.95 mL/min**	**R.T. (min)**	**Flow rate: 1.05 mL/min**	**R.T. (min)**

Injection 1	2.354	Injection 1	2.13
Injection 2	2.328	Injection 2	2.12
Injection 3	2.323	Injection 3	2.106
Average	2.335	Average	2.118666667
STD DEV	0.016643317	STD DEV	0.012055428
RSD (%)	0.712775888	RSD (%)	0.569010111

**pH 3.55 at 220 nm**	**R.T. (min)**	**pH 3.50**	**R.T. (min)**

Injection 1	2.83	Injection 1	2.177
Injection 2	2.84	Injection 2	2.179
Injection 3	2.82	Injection 3	2.186
Average	2.83	Average	2.180666667
STD DEV	0.01	STD DEV	0.004725816
RSD (%)	0.35335689	RSD (%)	0.21671426

**Caffeine**			
**Flow rate: 0.95 mL/min**	**R.T. (min)**	**Flow rate: 1.05 mL/min**	**R.T. (min)**

Injection 1	3.82	Injection 1	3.472
Injection 2	3.779	Injection 2	3.446
Injection 3	3.771	Injection 3	3.437
Average	3.79	Average	3.451666667
STD DEV	0.026286879	STD DEV	0.018175075
RSD (%)	0.693585194	RSD (%)	0.526559377

**pH 3.55 220 nm**	**R.T. (min)**	**pH 3.50**	**R.T. (min)**

Injection 1	4.83	Injection 1	3.591
Injection 2	4.87	Injection 2	3.609
Injection 3	4.82	Injection 3	3.577
Average	4.84	Average	3.592333333
STD DEV	0.026457513	STD DEV	0.016041613
RSD (%)	0.546642833	RSD (%)	0.446551338

**Naringin**			
**Flow rate: 0.95 mL/min**	**R.T. (min)**	**Flow rate: 1.05 mL/min**	**R.T. (min)**

Injection 1	4.791	Injection 1	4.515
Injection 2	4.789	Injection 2	4.507
Injection 3	4.781	Injection 3	4.515
Average	4.787	Average	4.512333333
STD DEV	0.005292	STD DEV	0.004618802
RSD	0.110539	RSD	0.102359507

**pH 3.55 220 nm**	**R.T. (min)**	**pH 3.50**	**R.T. (min)**

Injection 1	6.523	Injection 1	4.514
Injection 2	6.518	Injection 2	4.524
Injection 3	6.536	Injection 3	4.528
Average	6.525667	Average	4.522
STD DEV	0.009292	STD DEV	0.007211103
RSD	0.142385	RSD	0.159467106

^∗^The concentration was 31.25 (μg/mL) at 220 nm.

### 3.3. System Suitability

**FIGURE 4:**
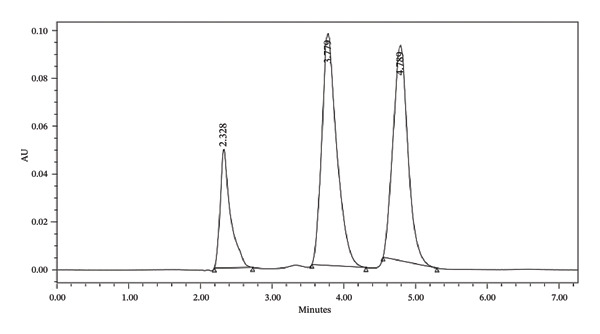
Chromatographic separation under the HPLC conditions of the developed method.

System suitability testing was performed to confirm that the chromatographic system was adequate for method validation by evaluating the tailing factor, theoretical plate count (*N*), and resolution (Rs) for the peaks of ascorbic acid, caffeine, and naringin (Figure 4). The tailing factors obtained were 1.38 for ascorbic acid, 1.317 for caffeine, and 1.507 for naringin, all within the acceptable limit of ≤ 2 as per validation guidelines. Theoretical plate counts were 2094.19 for ascorbic acid, 2292.16 for caffeine, and 3660.35 for naringin, all exceeding the minimum requirement of ≥ 2000. The resolution between ascorbic acid and caffeine was 5.820, and that between caffeine and naringin was 3.220, both above the acceptance criterion of > 2.5, indicating adequate separation. The detailed results are illustrated in Table 8, and these results demonstrate that the HPLC system met all system suitability criteria, confirming that it was suitable for the analysis during method validation [35].

**TABLE 8:** System suitability of the developed method.

*Tailing factor*	
Ascorbic acid	1.382037
Caffeine	1.316696
Naringin	1.506539

*Theoretical plate count* (*N*)	
Ascorbic acid	2094.185
Caffeine	2292.164
Naringin	3660.351

*Resolution* (*Rs*)	
Peak of ascorbic acid and caffeine	5.819572683
Peak of caffeine and naringin	3.219544846

### 3.4. Comparison of Developed Method With Reported HPLC Approaches

In recent studies, simultaneous detection methods have been established for various compounds in beverages, including ascorbic acid and caffeine. Notably, Turak et al. developed a robust RP‐UPLC method utilizing a Waters BEH C18 column, achieving successful separation of with a mobile phase composed of acetonitrile and 0.2 M H_3_PO_4_. Their detection wavelengths were 244 nm for ascorbic acid and 273.6 nm for caffeine, demonstrating excellent accuracy and precision [19]. However, the present method distinguishes itself by incorporating naringin alongside ascorbic acid and caffeine into a singular analytical run, a combination not covered in the existing literature. Furthermore, while the study employed dual‐wavelength detection, this approach enables detection at a single wavelength of 220 nm, ensuring balanced area percentages for all three compounds. This is not only simplifying the methodology but also enhancing accessibility. A systematic search of major scientific databases, including SciFinder and Web of Science, revealed no previously published methods reporting the simultaneous determination of ascorbic acid, caffeine, and naringin in a single chromatographic run. In contrast to the procedure by Turak et al., the isocratic method utilizing phosphate buffer and acetonitrile at ambient temperature in this study is validated across key parameters such as linearity, precision, and robustness. Additionally, Büyüktuncel reported an RP‐HPLC method for the simultaneous determination of naringin and hesperidin in citrus juices, identifying peaks at 280 nm [22]. While compatible in terms of naringin detection, their method lacks the inclusion of AA and CAF, emphasizing the comprehensive scope of the current methodology. The choice of a lower detection wavelength allows for the effective capture of all three analytes, contrasting with traditional flavonoid methods that typically operate at higher wavelengths [36]. This broader and more integrated approach highlights the potential of the presented method in beverage analysis.

## 4. Conclusion

The simultaneous quantification of ascorbic acid, caffeine, and naringin is of significant interest due to their therapeutic potential, particularly in colorectal cancer prevention and management. While numerous HPLC methods exist for individual determination, limited approaches address all three compounds in a single analytical run. This study aimed to develop and validate a robust RP‐HPLC method for its simultaneous analysis in accordance with ICH Q2(R1) and USP guidelines. Chromatographic separation was achieved using RP‐18 column with a mobile phase comprising phosphate buffer (pH 3.5), water, and acetonitrile. Detection was performed using a diode array detector at the optimal wavelength determined through comparative analysis. Method validation confirmed specificity, selectivity, linearity, precision, and accuracy recoveries. Robustness was demonstrated under deliberate variations in mobile phase composition, flow rate, and detection wavelength. The proposed method is reliable, reproducible, and suitable for routine quality control of formulations containing these bioactive compounds.

A robust RP‐HPLC method was developed and validated for the simultaneous determination of ascorbic acid, caffeine, and naringin in the concentration range of 7.813–125 μg/mL. Chromatographic separation was achieved on a Symmetry Shield RP‐18 column (250 × 4.6 mm, 5 μm) using an isocratic mobile phase of 75% phosphate buffer (pH 3.5) and 25% acetonitrile at a flow rate of 1 mL/min, with detection at 220 nm. Method validation confirmed specificity, selectivity, and excellent linearity (*R* ^2^ ≥ 0.9981). Precision testing showed RSD% values well below 2% for both intraday and interday analyses. Accuracy studies demonstrated mean recoveries within 98%–102% (ascorbic acid: 100.05%, caffeine: 100.33%, and naringin: 100.47%). Robustness testing indicated that small deliberate changes in the flow rate and pH had no significant effect on retention times (RSD% ≤ 0.71%). System suitability parameters, including tailing factor (≤ 1.5), theoretical plates (> 2000), and resolution (> 3.2), met the acceptance criteria. The developed method is reliable, reproducible, and suitable for routine quality control applications requiring synchronized quantification of these three bioactive compounds.

## Funding

No funding was received for this manuscript.

## Conflicts of Interest

The authors declare no conflicts of interest.

## Data Availability

The data supporting the findings of this study are available within the article and its Supporting Information.
